# PRED-TMSdeep: Prediction of Transmembrane Topology and Signal Peptides Using Deep Learning

**DOI:** 10.3390/biology15131016

**Published:** 2026-06-26

**Authors:** Grigorios A. Moschos, Konstantinos D. Tsirigos, Ioannis A. Tamposis, Pantelis G. Bagos

**Affiliations:** 1 Department of Biology, National and Kapodistrian University of Athens, 15701 Athens, Greece; grigmoschos@gmail.com; 2 The Novo Nordisk Foundation Biotechnology Research Institute for the Green Transition, Technical University of Denmark, DK-2800 Kongens Lyngby, Denmark; kots@dtu.dk; 3 Department of Computer Science and Biomedical Informatics, University of Thessaly, 35100 Lamia, Greece; itamposis@uth.gr

**Keywords:** deep learning, protein sequence annotation, transmembrane topology, signal peptide prediction

## Abstract

Accurate annotation of secreted and membrane proteins requires detecting N-terminal secretion signals, locating their cleavage sites, and distinguishing secretion-signal classes, while also predicting full transmembrane topology for both alpha-helical and beta-barrel proteins. Current tools typically address either whole-protein topology with a generic signal-peptide category or signal-peptide type classification without integrated topology annotation, leaving end-to-end labels incomplete when both features must be resolved together. Here, we present PRED-TMSdeep, a deep learning method that jointly predicts transmembrane topology and three signal peptide classes: secretory pathway/signal peptidase I (Sec/SPI), secretory pathway/signal peptidase II (Sec/SPII), and twin-arginine translocation/signal peptidase I (Tat/SPI). We introduce a two-step constrained decoding procedure that first detects transmembrane segments and signal peptides and then resolves global orientation and refines boundaries under stricter biological constraints. On redundancy-reduced datasets curated from the Orientation of Proteins in Membranes and the Protein Data Bank of Transmembrane Proteins, PRED-TMSdeep matches leading predictors for segment-level topology while improving signal peptide classification and yielding the highest overall top-1 cleavage-site accuracy. Top-1 cleavage-site accuracy reached 89.2%, compared with 84.7% for TMbed and 86.2% for SignalP 6.0, mainly reflecting strong performance on the predominant Sec/SPI class. The software is available as a web server and a batch command-line tool with pretrained models and reproducible workflows.

## Simple Summary

Proteins that are secreted from cells or embedded in cell membranes are essential for communication, transport of nutrients, and many medical and industrial applications. To study these proteins, scientists first need to know whether a protein contains an “address tag” at its beginning that sends it to the secretion machinery, and whether it also contains parts that cross the membrane. These two features can look similar in the sequence, so prediction tools often confuse them or provide incomplete annotations. We present PRED-TMSdeep, a deep learning tool that predicts, in one step, both membrane-crossing regions (including two major membrane protein types) and three biologically different classes of secretion signals, together with the most likely cleavage site where the signal is removed. Using carefully curated reference datasets derived from experimentally determined membrane protein structures and protein databases, the method matches leading tools for membrane topology while improving the identification of secretion signal classes and their cleavage sites. PRED-TMSdeep is provided as an easy-to-use web server and a batch command-line tool for large datasets, supporting routine protein annotation and large-scale genome and proteome studies.

## 1. Introduction

Transmembrane proteins (TMPs) are central to many essential cellular processes, including signaling, transport, secretion, and enzymatic activity [1]. These proteins are embedded in biological membranes through hydrophobic transmembrane segments that typically adopt either α-helical or β-barrel conformations. Their biological role is closely linked to their membrane topology, i.e., the number and positions of membrane-spanning regions and their orientation relative to the intracellular and extracellular environments [2]. Accurate topology annotation is therefore important for understanding protein function, molecular interactions, and cellular localization.

Experimentally, membrane protein topology can be inferred from three-dimensional structures determined by methods such as X-ray crystallography or cryo-electron microscopy and subsequently positioned in the lipid bilayer using dedicated computational tools, including PPM and TMDET [3,4,5,6]. Despite their value, these experimental approaches remain difficult to apply broadly because membrane proteins are challenging to solubilize, purify, and crystallize [7]. As a result, TMPs remain underrepresented in the Protein Data Bank compared with soluble proteins [8], limiting the availability of experimentally validated structural annotations.

To address this limitation, a wide variety of computational methods have been developed to predict membrane topology directly from amino acid sequences. Early predictors relied mainly on hydrophobicity profiles and sliding-window analysis, whereas later methods introduced probabilistic sequence models such as hidden Markov models (HMMs), substantially improving topology prediction accuracy [9,10,11,12]. Subsequent methods incorporated signal peptide recognition into topology prediction, since signal peptides and transmembrane helices share similar hydrophobic properties and are therefore often confused [13,14,15]. In parallel, dedicated signal peptide predictors were developed to identify secretion signals and distinguish among different secretion-related classes [16,17,18].

This distinction is biologically important because signal peptides are short N-terminal targeting sequences that direct proteins to membrane translocation pathways such as Sec or Tat. Although they are not transmembrane domains in the structural sense, their hydrophobic core regions resemble membrane-spanning helices, making discrimination difficult when the two problems are treated separately. Joint modeling of membrane topology and signal peptides can therefore reduce false positives and improve the biological consistency of protein annotations.

More recently, deep learning methods have further advanced the field. Predictors such as DeepTMHMM 1.0 and TMbed exploit contextual protein representations derived from large-scale sequence modeling and have achieved strong performance in topology prediction [19,20]. At the same time, modern versions of SignalP have markedly improved the classification of signal peptide types and the localization of cleavage sites [21,22,23]. However, current methods still tend to address these tasks only partially. Topology predictors generally provide whole-protein membrane annotation but usually treat signal peptides as a single generic category, whereas signal peptide predictors distinguish among secretion signal types without resolving complete membrane topology. Consequently, a unified framework capable of jointly predicting transmembrane topology together with explicit signal peptide classes, including secretory pathway/signal peptidase I (Sec/SPI), secretory pathway/signal peptidase II (Sec/SPII), and twin-arginine translocation/signal peptidase I (Tat/SPI), is still lacking.

In this study, we present PRED-TMSdeep, a deep learning framework designed to address this gap by jointly predicting transmembrane topology and signal peptide type for both α-helical and β-barrel membrane proteins. To support this task, we assembled a high-confidence dataset by integrating membrane annotations from the Orientation of Proteins in Membranes database and the Protein Data Bank of Transmembrane Proteins [24,25]. We then extended the TMbed architecture with an expanded output space that explicitly models distinct signal peptide classes and introduced a constrained two-step decoding strategy that separates local segment detection from global topology inference. This design enables robust transmembrane segment prediction while improving signal peptide classification and cleavage-site localization. Evaluated using nested cross-validation, PRED-TMSdeep provides more complete protein annotations by combining transmembrane topology and signal peptide type within a single prediction framework.

## 2. Materials and Methods

The proposed method, PRED-TMSdeep, jointly predicts transmembrane topology and signal peptide type from amino acid sequences using a unified probabilistic framework. The approach integrates residue-level posterior estimation with biologically constrained decoding to produce globally consistent annotations of membrane-spanning regions, signal peptide classes, cleavage positions, and protein orientation. In the following subsections, we describe the problem formulation, the learning of the posterior model, the construction of the decoding graph, the two-step dynamic-programming decoding procedure, the integration of prior information, the complete prediction pipeline, the evaluation criteria, and the software implementation of the web server and batch command-line tool.

### 2.1. Problem Formulation

Let $X=\left(x_{1},\ldots ,x_{L}\right)$ be an amino acid sequence of length L and let $\pi =\left(\pi_{1},\ldots ,\pi_{L}\right)$ denote its residue-level topology, where $\pi_{i}\in A=\{S,\;L,\;T,\;H,\;B,\;I,\;O\}$ corresponding to Sec/SPI, Sec/SPII, Tat/SPI, alpha-helical transmembrane segment, beta-barrel transmembrane strand, inside, and outside, respectively. Our goal is to predict the most probable topology given the amino acid sequence which is defined by the following Equation (1).

(1) $$\pi^{*}=\arg\underset{\pi }{\max}P\left(\left.\pi \right|X\right)$$

To enforce biological constraints, decoding is performed in an expanded state space S, whose states refine the residue labels to encode topology and segment-length restrictions. The biological constraints are represented by a directed decoding graph over S, defined by a transition indicator function $\delta \left(s_{i},s_{i+1}\right)\in \{0,\;1\}$, where $\delta \left(s_{i},s_{i+1}\right)=1$ if $s_{i}\to s_{i+1}$ is an allowed edge, together with start and end indicators $b(s_{1})\in \{0,1\}$ and $e(s_{L})\in \{0,1\}$, which specify whether a state is permitted as the first or last state of a valid path.

A surjective map g:S→A projects each expanded state to its corresponding residue label. The function $P_{\theta }\left(\left.\pi_{i}\right|X\right)$ denotes the posterior probability of label $\pi_{i}$ at position i. The resulting constrained optimization problem is therefore formulated as given in Equation (2).

(2) $$\hat{s}=\arg\underset{s_{1,}s_{2},\ldots ,s_{L}}{max}b(s_{1})e(s_{L})\prod_{i=1}^{L-1}\delta \left(s_{i},s_{i+1}\right)\prod_{i=1}^{L}P_{\theta }\left(\left.\pi_{i}=g\left(s_{i}\right)\right|X\right)$$

The final topology sequence is then recovered elementwise as shown in Equation (3).

(3) $$\hat{\pi_{i}}=g\left(\hat{s_{i}}\right),\;\;\;\;\;\;\;i=1,\ldots ,L$$

This formulation is related to Posterior Viterbi decoding [26], except that here the per-position posteriors are produced by a neural network rather than by an HMM. In the remainder of this section, we describe how $P_{\theta }\left(\left.\cdot \;\right|X\right)$ is learned, define the decoding graph, and present the dynamic-programming decoding algorithm.

### 2.2. Learning the Posterior Model

To learn the posterior model $P_{\theta }\left(\left.\cdot \;\right|X\right)$, we first construct the training data, then define the TMbed-based neural network, and finally describe the training and evaluation protocol.

#### 2.2.1. Data Collection and Preprocessing

**Figure 1:**
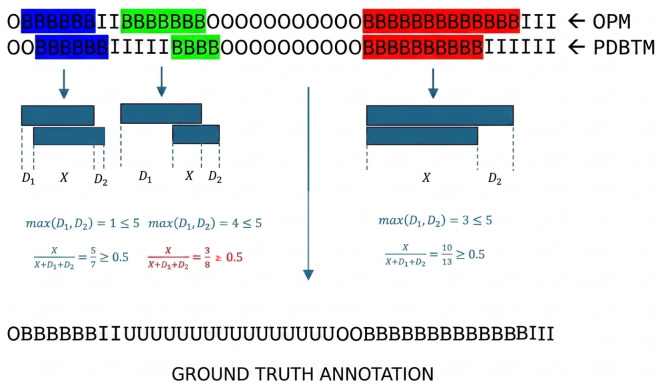
Example construction of a binary OPM–PDBTM agreement mask. The figure shows how, for each transmembrane (TM) segment, we decide whether the OPM annotation is transferred to the dataset. At the top, the first two tracks display TM labels from OPM (top) and PDBTM (bottom). Arrows highlight three example segments; for each, we compute the overlap X and the boundary deviations D_1_ (start) and D_2_ (end). For the first segment (blue), both criteria are satisfied, so mask = 1 and the annotation is transferred. For the second (green), the overlap constraint is not met, so mask = 0 and the segment is relabeled “U” and ignored during training. For the third (red), both criteria are again satisfied, so mask = 1 and the annotation is transferred. The bottom track shows the resulting sequence. In this example the mask is [1, 0, 1], allowing the model to train only on high-confidence TM regions.

We assembled transmembrane (TM) and non-transmembrane (non-TM) datasets from publicly available resources. For TM proteins, we intersected OPM and PDBTM, mapped the overlapping entries to UniProt [27] via SIFTS [28,29], and retained only high-confidence TM segments for which the two resources agreed, defined by an intersection-over-union (IoU) of at least 0.5 (see Equation (S2), Supplementary Materials) and start/end boundary differences of no more than five residues (see Equation (S1), Supplementary Materials). Agreed segments were used as training labels, whereas non-agreeing segments were relabeled as unknown (U) and ignored during training, as shown in Figure 1. Additional details on the agreement procedure are provided in the Supplementary Material. Proteins for which all annotated TM segments satisfied these agreement criteria were considered fully trusted, whereas proteins containing a mixture of agreeing and non-agreeing segments were considered partially trusted. After redundancy reduction with CD-HIT [30,31] at 20% sequence identity and length filtering to 50–6000 residues, the TM set comprised 718 alpha-helical and 82 beta-barrel proteins.

For the non-TM set, we combined signal peptide (SP)-positive sequences from SignalP, mapped back to full-length UniProt entries, with globular non-TM proteins from TMbed, and reclustered the merged set at 20% identity. We then removed any sequence sharing at least 40% identity with OPM-derived TM entries or annotated with TM regions in UniProt, yielding 6293 proteins: 5059 without SPs, 881 Sec/SPI, 60 Tat/SPI, and 293 Sec/SPII proteins.

Finally, TM segments failing the OPM–PDBTM agreement criteria were excluded from training and retained as a separate low-confidence independent test set. This procedure allowed us to train on high-confidence annotations while preserving an independent benchmark for evaluating robustness under annotation ambiguity.

#### 2.2.2. Model Architecture

Our method builds upon TMbed to jointly predict transmembrane topology and three signal peptide classes: Sec/SPI, Sec/SPII, and Tat/SPI. The architecture follows the original TMbed design with two modifications.

- First, the final output layer is expanded from five to seven classes, enabling explicit prediction of Sec/SPI, Sec/SPII and Tat/SPI; the convolutional neural network (CNN) backbone remains unchanged.
- Second, we introduce a beta-barrel refinement stage in which proteins initially predicted as beta-barrel by the primary model are reprocessed by a second CNN trained exclusively on beta-barrel sequences. This refinement network predicts only the B, I, and O classes and applies a separate decoding process, while the signal peptide predictions from the first stage are retained unchanged.

#### 2.2.3. Training and Evaluation Protocol

We adopted the nested cross-validation protocol of TMbed to ensure robust model selection and unbiased performance estimation. The dataset was stratified by protein type and signal peptide status and distributed evenly across five folds (Supplementary Text S1, Supplementary Materials). In each outer split, one fold was used for testing, while the remaining folds were divided into training and validation sets for model selection (Supplementary Text S1, Supplementary Materials). After selecting the number of training epochs based on validation performance, the model was retrained on the combined training and validation data and evaluated on the held-out test fold. The same outer-fold partitioning was used for both the primary joint model and the beta-barrel refinement model, and final performance was obtained by aggregating predictions across the five test folds. Both models were trained with the AdamW [32] optimizer, with the refinement model trained only on the beta-barrel subset of each split. Full details of the data stratification, fold assignment, nested cross-validation procedure, and training settings are provided in Supplementary Text S1 (Supplementary Materials).

### 2.3. Construction of the Decoding Graph

**Figure 2:**
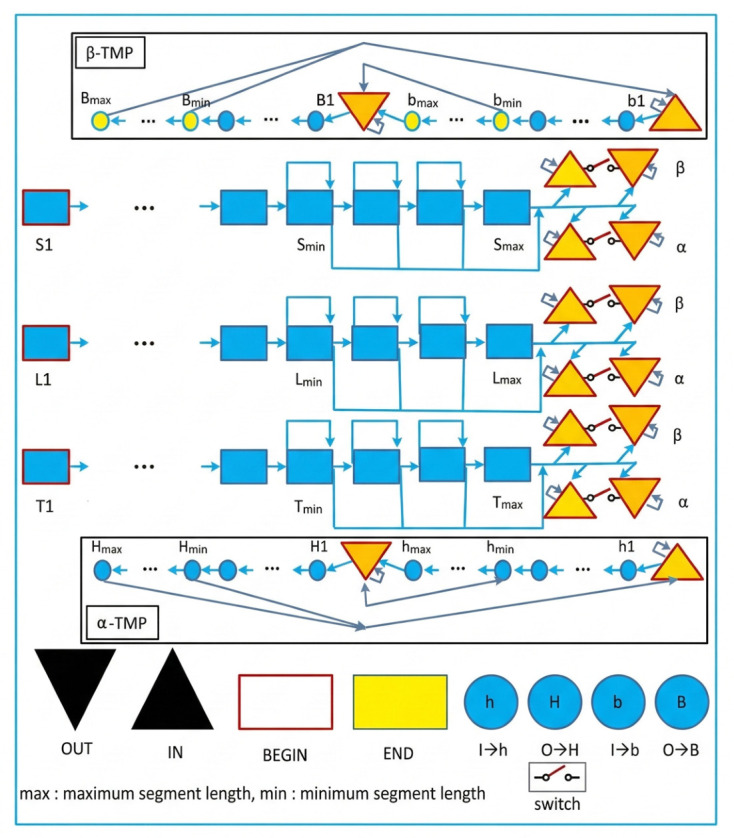
Directed decoding graph used by PRED-TMSdeep. The graph contains separate paths for Sec/SPI (S), Sec/SPII (L), and Tat/SPI-related (T) signal peptides, as well as states for α-helical transmembrane segments (H), β-barrel strands (B), and inside/outside loop regions. Signal peptide, helix, and strand paths encode class-specific length constraints. Ellipses (…) indicate repeated intermediate states within length-constrained paths. Triangles denote loop states, with IN and OUT indicating the two possible loop orientations. Arrows indicate allowed transitions through the graph; arrow colors are used only for visual clarity and do not denote different transition types. The switch symbol represents whether direct inside-to-outside and outside-to-inside transitions are allowed. During the first Viterbi pass, the switch is closed, allowing relaxed topology constraints to prioritize structured-region detection. During the second pass, the switch is open, direct I ↔ O transitions are disallowed, and the final global topology is resolved under stricter membrane-crossing constraints.

We next define the decoding graph that specifies the valid state paths used by the decoder, as illustrated in Figure 2. As in TMbed, the residue labels are refined into an expanded state space so that biological constraints can be enforced directly through graph connectivity. However, our graph differs from that of TMbed in three respects: it models three distinct signal peptide classes (S, L, and T) instead of a single SP class, it uses label-specific segment-length constraints derived from empirical distributions, and it includes explicit switches that allow alternative topology configurations to be represented within the graph.

For each structured class, the graph contains ordered substates corresponding to progression through a segment. Thus, alpha-helical segments (H), beta-barrel strands (B), and the three signal peptide classes (S, L, and T) are represented by class-specific state chains, together with begin/end states and inside (I) and outside (O) states for non-structured regions. Unlike TMbed, which enforces a uniform minimum length of five residues for SP and TM segments, we use biologically motivated label-specific constraints fixed a priori from empirical length histograms: $6\leq \left|B\right|\leq 15$, $11\leq \left|H\right|\leq 29$ and minimum lengths of 13, 14, and 22 residues for Sec/SPI, Sec/SPII, and Tat/SPI, respectively. The signal peptide classes additionally include self-loops in their terminal substates to accommodate longer right-tailed length distributions, whereas H and B segments use bounded ranges without self-loops.

Finally, the graph includes switches between inside and outside states to represent two decoding regimes: with the switch closed, the topology constraints are relaxed and direct transitions between I and O are allowed, whereas with the switch open, the constraints are stricter and transitions between I and O are only allowed through an intervening transmembrane segment. Overall, the proposed graph retains the grammar-based decoding principle of TMbed but extends it to support explicit multi-class signal peptide modeling and segment-length constraints derived from empirical class-specific length distributions.

### 2.4. Two-Step Dynamic-Programming Decoding

The decoding procedure separates the prediction problem into two related subproblems: detection of structured regions and resolution of a biologically valid global topology. This separation is useful because segment detection and topology consistency impose different constraints. A single strict decoding step must optimize both simultaneously, which can force the decoder to alter or split otherwise plausible transmembrane regions to satisfy global inside/outside constraints. Therefore, PRED-TMSdeep first identifies structured regions under relaxed topology constraints and then resolves the final orientation and inside/outside topology under stricter constraints. Given the posterior probabilities $P_{\theta }\left(\left.\cdot \;\right|X\right)$ and the decoding graph in Figure 2, the constrained optimization problem in Equation (2) is solved by dynamic programming using a two-step Viterbi decoding procedure. In the first step, the switches are closed, and the first-stage decoder $V_{1}$ focuses on segment detection, predicting transmembrane segments (H, B) and signal peptide classes (S, L, T) while allowing transitions between inside (I) and outside (O) states. This relaxed topology grammar follows the rationale of TMbed: if direct I ↔ O transitions are prohibited from the start, the decoder may be forced to split an otherwise continuous transmembrane segment by inserting a loop state in order to satisfy strict topology constraints. Thus, the first pass prioritizes the detection of structured regions without requiring the temporary topology to be globally consistent. Positions predicted as structured regions (i.e., positions assigned to classes S, L, T, H, or B) are then fixed by replacing their posterior probabilities with one-hot encodings, so that confident local predictions are preserved in the next stage.

In the second step, the switches are open and the second-stage decoder $V_{2}$ resolves the global topology under stricter structural constraints. The original probability matrix is first smoothed by temperature scaling at non-fixed positions (i.e., positions assigned to classes I or O), after which a conditional merge combines the fixed structured predictions from $V_{1}$ with the smoothed probabilities at the remaining positions. This smoothing reduces the influence of overly confident local I/O assignments and allows the second decoder to adjust loop labels while preserving the structured regions detected in the first pass. Decoder $V_{2}$ then selects the N-terminal orientation and enforces consistent membrane-crossing behavior across the sequence, disallowing direct transitions between inside and outside states. In this way, the first pass emphasizes accurate detection of structured regions, whereas the second pass produces a globally consistent topology. Algorithm 1 summarizes the complete two-step decoding procedure.
**Algorithm 1:** Two-step Viterbi decoding procedure
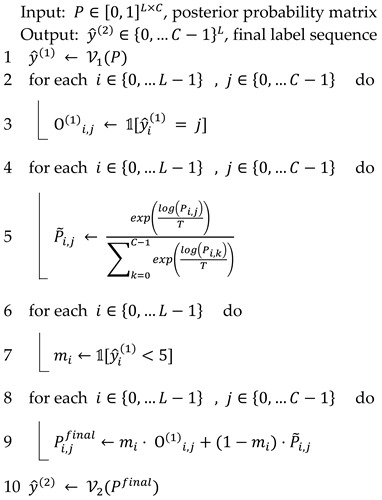


**Algorithm 1**: The algorithm summarizes the two-step decoding process used in PRED-TMSdeep. First, the segment-level decoder $V_{1}$ predicts structured regions from the input probabilities $P\in {\left[0,\;1\right]}^{L\times C}$ where L is the sequence length and C=7 is the number of output classes. These predictions are converted to one-hot labels. A softmax operation with fixed temperature T=3.5 is then applied to smooth the original probabilities. A conditional merge combines the fixed and smoothed outputs (where “fixed” refers to positions predicted as structured classes S, L, T, H, and B (labels 0–4) by the first-step decoder and replaced with one-hot encodings). Finally, the topology-level decoder $V_{2}$ generates the final label sequence with global structural constraints. Unlike the first decoder, this stage does not allow arbitrary I ↔ O transitions (with the switch open).

### 2.5. Sensitivity Analysis of the Temperature Parameter

The temperature scaling in Algorithm 1 is applied only before the second Viterbi decoding step and only at positions that were not fixed as structured regions after the first decoding pass. Therefore, temperature scaling primarily affects the loop regions used to resolve the final topology, although small adjustments at the boundaries of transmembrane regions may still occur during the second decoding step if supported by the posterior probabilities and the decoding constraints.

The rationale for this smoothing is related to the role of the second decoding step. After the first pass, the structured regions detected with high confidence are preserved, and the second decoder resolves the global inside/outside topology under stricter constraints. If the posterior probabilities at non-fixed I/O positions remain too sharp, the decoder may be overly constrained by local loop-label assignments and may fail to recover the most consistent global topology. For example, if the neural network assigns high probability to the same loop label on both sides of a predicted transmembrane segment, the strict topology grammar may resolve this inconsistency by altering or splitting the transmembrane segment instead of changing one of the loop labels. By softening the probabilities at non-fixed positions, temperature scaling allows the second Viterbi pass to adjust surrounding I/O labels more easily while preserving the structured regions detected in the first pass.

**Figure 3:**
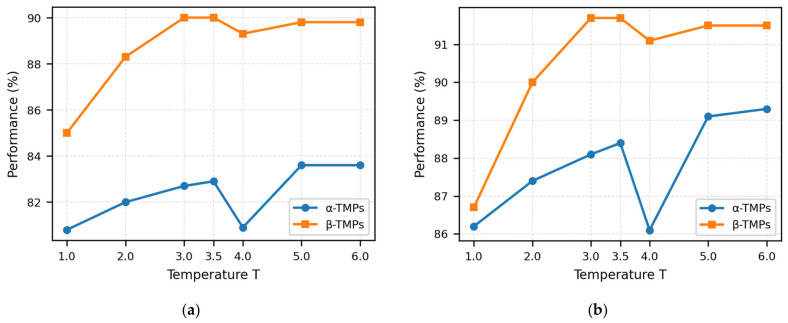
Sensitivity analysis of the temperature parameter T applied before the second Viterbi decoding step. The temperature parameter is applied only to non-fixed positions after the first decoding pass, while positions predicted as structured regions are kept fixed. T = 1 corresponds to no temperature smoothing, whereas T > 1 softens the posterior probabilities at non-fixed inside/outside positions to facilitate resolution of the global topology under stricter constraints. (**a**) Effect of T on correct topology prediction for alpha-helical and beta-barrel transmembrane proteins. (**b**) Effect of T on prediction of the correct number of transmembrane segments for the same protein classes. In both panels, increasing T above 1 improves topology-level consistency, with T = 3.5 providing balanced improvement for both alpha-helical and beta-barrel proteins.

To evaluate the effect of T, we performed a sensitivity analysis over different temperature values and focused on the topology-level metrics most directly affected by the second decoding step: correct topology and correct number of transmembrane segments. The case T = 1 corresponds to leaving the posterior probabilities unchanged, i.e., applying no temperature smoothing. As shown in Figure 3a, using T > 1 improved correct topology compared with T = 1. For alpha-helical transmembrane proteins, correct topology increased from 80.8% at T = 1 to 82.9% at T = 3.5. For beta-barrel transmembrane proteins, the corresponding improvement was from 85.0% to 90.0%.

A similar trend was observed for the correct number of transmembrane segments, as shown in Figure 3b. For alpha-helical transmembrane proteins, this metric increased from 86.2% at T = 1 to 88.4% at T = 3.5, whereas for beta-barrel transmembrane proteins it increased from 86.7% to 91.7%. These results support the use of temperature smoothing before the second decoding step, since its main effect is to improve global topology consistency. The value T = 3.5 was retained as a fixed smoothing value because it provided a balanced improvement for both alpha-helical and beta-barrel topology metrics. We do not treat this value as a finely optimized hyperparameter; rather, the analysis shows that temperature smoothing itself is beneficial and that T = 3.5 lies in a stable range with improved topology-level performance relative to T = 1.

### 2.6. Relationship to TMbed and Novel Components

Before extending the prediction task, we first reimplemented the TMbed decoding framework on the original TMbed dataset and examined whether the Viterbi transition scheme could be improved without modifying the underlying convolutional neural network architecture. In TMbed, transmembrane segments and signal peptides are constrained using a uniform minimum length of five amino acids with self-loops. We replaced this rule with label-specific minimum and maximum length constraints derived from empirical length distributions of signal peptide classes, α-helical transmembrane segments, and β-barrel strands. In the TMbed reimplementation, this modification improved several standard TMbed evaluation metrics. For α-helical transmembrane proteins, recall and precision increased from 88.5% and 88.8% to 89.2% and 89.6%, respectively, with Qok and Qnum increasing by approximately 1–2 percentage points and Qtop remaining unchanged at 96.5%. For β-barrel transmembrane proteins, recall increased from 95.3% to 95.4%, precision from 99.2% to 99.3%, and Qok from 80.5% to 82.0%, whereas Qnum remained unchanged at 88.1% and Qtop decreased slightly from 97.7% to 97.1%. These results motivated the use of label-specific length constraints in PRED-TMSdeep.

Building on this modified TMbed framework, PRED-TMSdeep extends the prediction task to seven residue-level classes, enabling simultaneous prediction of transmembrane topology and three signal peptide classes, Sec/SPI, Sec/SPII, and Tat/SPI. To preserve topology performance in this expanded task and on the newly curated OPM–PDBTM dataset, we introduced two additional components. First, a two-step Viterbi decoding procedure detects structured regions under relaxed topology constraints and then resolves the final orientation under stricter constraints as described above. Second, proteins classified as β-barrel transmembrane proteins are reprocessed by a β-barrel-specific refinement convolutional neural network, while signal peptide predictions from the first model are retained. Thus, PRED-TMSdeep inherits the TMbed neural backbone and general decoding concept, but adapts them through label-specific length constraints, multi-class signal peptide modeling, two-step decoding, and β-barrel-specific refinement.

### 2.7. Prior Information Integration

In practical applications, partial prior topological information may be available from experiments, domain knowledge, or manual curation, and incorporating such information can improve consistency and accuracy. This idea has been explored previously in HMM-based topology prediction, where fixing parts of the sequence to known labels was shown to improve constrained predictions when reliable external information is available [33]. In our framework, prior information is incorporated before dynamic programming by overwriting the posterior probability matrix produced by the CNN at user-specified positions. Specifically, if the user provides a label assignment for an interval, the corresponding posterior vectors are replaced with fixed label encodings before decoding. The modified posterior matrix is then passed unchanged to the decoder, allowing the final prediction to remain consistent with the supplied prior information while guiding the construction of the overall topology.

### 2.8. End-to-End Prediction Pipeline

The proposed pipeline operates end-to-end from an input amino-acid sequence in FASTA format. The primary CNN first produces residue-level probabilities over {S, L, T, H, B, I, O}, which are decoded using the two-step Viterbi algorithm. Non-TM and alpha-TMP predictions are returned directly. For predicted beta-TMPs, the original sequence is reprocessed by the beta-barrel CNN to obtain refined probabilities over {B, I, O}. The signal peptide probabilities {S, L, T} from the primary CNN are then transferred to the refined probability matrix, and the decoding step is repeated to produce the final prediction.

### 2.9. Evaluation Criteria

We evaluated performance at three levels: signal peptide prediction, segment-level prediction, and protein-level classification, reporting alpha-helical and beta-barrel transmembrane proteins separately where appropriate. Signal peptide performance was assessed by type, by presence/absence, and by top-k cleavage-site accuracy, with k = 1 and k = 3 corresponding to predictions within 1 and 3 residues of the annotated cleavage site, respectively. Segment-level evaluation included segment overlap (SOV) [34], residue-level accuracy (Q3), correct topology, correct number of transmembrane segments, and precision/recall as in TMbed; precision and recall were computed on both fully and partially trusted proteins, whereas all other segment-level metrics were restricted to fully trusted proteins. Correct topology was defined by comparing compressed predicted and reference label sequences obtained by collapsing consecutive identical labels (e.g., IIIIBBBBOOO becomes IBO), so that a prediction was considered correct only if both the number of transmembrane segments and the N-terminal orientation were recovered. At the protein level, recall and false-positive rate (FPR) were computed for each structural class (alpha-helical TMP, beta-barrel TMP, and non-TM) from the confusion matrix aggregated across all folds.

### 2.10. Web Server and Command-Line Implementation

PRED-TMSdeep is provided both as a web server for interactive use and as a batch command-line Python tool for large-scale analyses. The command-line tool was implemented in Python 3.10, and the source code corresponds to the version described in this study. In both implementations, prediction is performed using the ensemble of the five models described in this study. For each input sequence, residue-level posterior probabilities are first obtained from all five models, averaged to generate a consensus posterior estimate, and then decoded to produce the final topology annotation. Thus, both implementations follow the same end-to-end prediction pipeline described in the previous subsections.

The web server, available at https://hannibal.dib.uth.gr/PRED-TMSdeep/ (accessed on 22 June 2026), accepts one protein sequence at a time in standard FASTA format and returns complete per-residue annotations, including transmembrane topology, signal peptide class, cleavage-site prediction, and global orientation. It is intended for interactive small-scale analysis and also allows the user to provide prior topological information when available. Because the web server processes one sequence per submission, large FASTA files or proteome-scale analyses should be performed with the batch command-line implementation. The command-line version enables high-throughput analysis of large protein datasets using the same pretrained ensemble and decoding framework.

## 3. Results

All results are reported by aggregating predictions across the five independent outer test folds of the nested cross-validation protocol, using the evaluation criteria defined in Section 2.9. To quantify uncertainty, we additionally estimated 95% confidence intervals by bootstrap resampling at the protein level, rather than at the residue or segment level, so that within-protein dependence was preserved. Bootstrap confidence intervals were calculated for the main signal peptide benchmarks, including the comparison with TMbed and SignalP 6.0, and for the PRED-TMSdeep topology results on the full α-TMP and β-TMP evaluation sets. We also evaluate a separate low-confidence independent test set comprising TM segments excluded from training due to annotation disagreement between OPM and PDBTM. Comparisons with prior methods followed a common protocol. For TMbed, SignalP 6.0 and DeepTMHMM 1.0, we used prediction outputs obtained from the respective sources, mapped them to our identifiers, and retained only proteins present in both datasets. All comparative analyses were performed on these common subsets and are reported separately from results on the full evaluation sets.

### 3.1. Signal Peptide Prediction Performance

**Figure 4:**
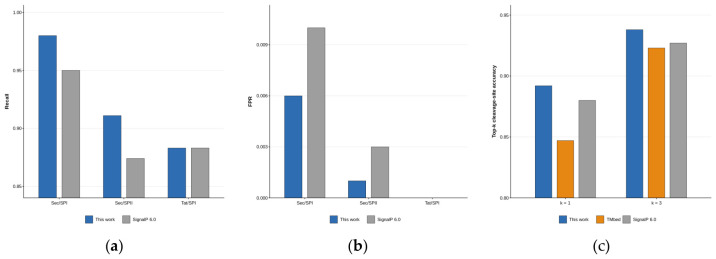
Signal peptide prediction performance of PRED-TMSdeep compared with TMbed and SignalP 6.0 on the full signal peptide evaluation dataset. The evaluation set contained 6293 proteins, including 1234 signal peptide-positive proteins: 881 Sec/SPI, 293 Sec/SPII, and 60 Tat-related signal peptides, together with 5059 non-signal-peptide proteins. (**a**) Class-wise recall for Sec/SPI, Sec/SPII, and Tat/SPI signal peptides. (**b**) Class-wise false positive rate (FPR) for the same signal peptide classes. (**c**) Overall top-k cleavage-site accuracy for k = 1 and k = 3. For consistency with the PRED-TMSdeep label space, SignalP 6.0 Tat/SPI and Tat/SPII predictions were merged into the Tat/SPI-related class. Cleavage-site accuracy was calculated only for true-positive signal peptide predictions of the corresponding class. Values are shown as fractions, with 1.0 corresponding to 100% performance.

PRED-TMSdeep improved signal peptide prediction by explicitly modeling the three biologically distinct classes Sec/SPI, Sec/SPII, and Tat/SPI, rather than treating signal peptides as a single generic category. This class-aware formulation enabled the model to capture type-specific sequence patterns and supported more accurate discrimination among heterogeneous signal peptide classes. As summarized in Figure 4, PRED-TMSdeep showed stronger recall than the competing methods for Sec/SPI and Sec/SPII, while maintaining performance comparable to SignalP 6.0 for Tat/SPI. At the same time, false positive rates remained low across all classes, indicating that the improved sensitivity was not achieved at the expense of reduced specificity. Bootstrap confidence intervals were calculated by resampling proteins and are reported for the main signal peptide metrics to quantify the uncertainty of these estimates.

The overall comparison of cleavage-site localization also favored PRED-TMSdeep. As shown in Table 1, the method achieved the highest overall top-1 cleavage-site accuracy, reaching 89.2%; the corresponding bootstrap confidence interval was [87.4, 90.9]. The corresponding point estimates were 84.7% for TMbed and 86.2% for SignalP 6.0, with bootstrap confidence intervals of [82.5, 87.5] and [84.3, 88.1], respectively. A similar trend was observed for top-3 accuracy, where PRED-TMSdeep reached 93.8% [92.4, 95.2], compared with 92.3% [90.0, 94.5] for TMbed and 91.8% [90.2, 93.4] for SignalP 6.0. These bootstrap intervals indicate that the overall cleavage-site estimates are stable, although modest differences between methods should be interpreted cautiously because the intervals quantify uncertainty for each method separately rather than formal paired statistical significance. Thus, explicit signal peptide class modeling improved the overall localization of cleavage positions while preserving strong detection performance.

A more detailed class-specific comparison is provided in Table 1, with the corresponding 95% bootstrap confidence intervals reported in Supplementary Table S1. For Sec/SPI signal peptides, PRED-TMSdeep showed higher recall than SignalP 6.0, achieving 98.0% compared with 93.1%. The corresponding bootstrap intervals, 97.0–98.9% and 91.3–94.7%, respectively, support a robust improvement in Sec/SPI sensitivity. The difference in top-1 cleavage-site accuracy was smaller, 90.5% versus 88.4%, and should be interpreted more cautiously because the corresponding intervals partially overlap.

For Sec/SPII signal peptides, PRED-TMSdeep showed favorable point estimates for recall, false positive rate, and cleavage-site localization in Table 1. However, the confidence intervals reported in Supplementary Table S1 indicate that several of these differences should not be overinterpreted.

For Tat/SPI signal peptides, both methods achieved identical recall, whereas SignalP 6.0 showed higher cleavage-site accuracy. The wider Tat/SPI intervals in Supplementary Table S1 reflect the small number of Tat/SPI proteins. Therefore, the higher overall cleavage-site localization observed for PRED-TMSdeep mainly reflects strong Sec/SPI performance, with favorable point estimates for Sec/SPII, whereas Tat/SPI remains the main class-specific limitation.

Overall, these results show that PRED-TMSdeep provides a favorable balance between signal peptide classification and cleavage-site localization within a unified topology-prediction framework. The method was particularly strong for Sec/SPI and Sec/SPII prediction, while Tat/SPI remained the most challenging signal peptide class.

### 3.2. Protein Classification Performance

Our method preserves the strong protein-level classification performance of TMbed while operating on an expanded dataset. Our updated dataset includes 718 alpha-helical TMPs, 82 beta-barrel TMPs, and 6293 non-transmembrane proteins, compared to 593, 65, and 5654, respectively, in TMbed. Classification accuracy remains stable across all three classes, with recall and false positive rates (FPR) of 0.985/0.004 for alpha-helical TMPs, 0.939/0.007 for beta-barrel TMPs, and 0.988/0.018 for non-transmembrane proteins.

### 3.3. Segment-Level Prediction Performance

We report segment-level results for both alpha-helical and beta-barrel TMPs. For each class, (i) this work was evaluated on the full dataset, and (ii) a direct comparison with TMbed and DeepTMHMM 1.0 was performed on the subset of proteins common to each pair of methods.

On the full alpha-helical dataset (718 proteins), our method attains SOV = 0.924 [0.909, 0.939], Q3 = 0.886 [0.871, 0.899], correct topology = 0.829 [0.797, 0.859], correct number of TM segments = 0.884 [0.857, 0.909], precision = 0.89 [0.874, 0.905] and recall = 0.914 [0.902, 0.926]. On the full beta-barrel dataset (82 proteins), PRED-TMSdeep reached SOV = 0.962 [0.927, 0.988], Q3 = 0.926 [0.901, 0.946], correct topology = 0.900 [0.820, 0.967], correct number of TM segments = 0.917 [0.840, 0.982], precision = 0.955 [0.919, 0.983], and recall = 0.938 [0.889, 0.977]. These confidence intervals show that the topology estimates are generally stable, especially for the larger alpha-helical dataset. As expected, the beta-barrel intervals are wider because this subset contains only 82 proteins. Therefore, the beta-barrel results should be interpreted with appropriate caution, but they nevertheless indicate that PRED-TMSdeep maintains strong segment-level topology performance on both alpha-helical and beta-barrel TMPs.

**Figure 5:**
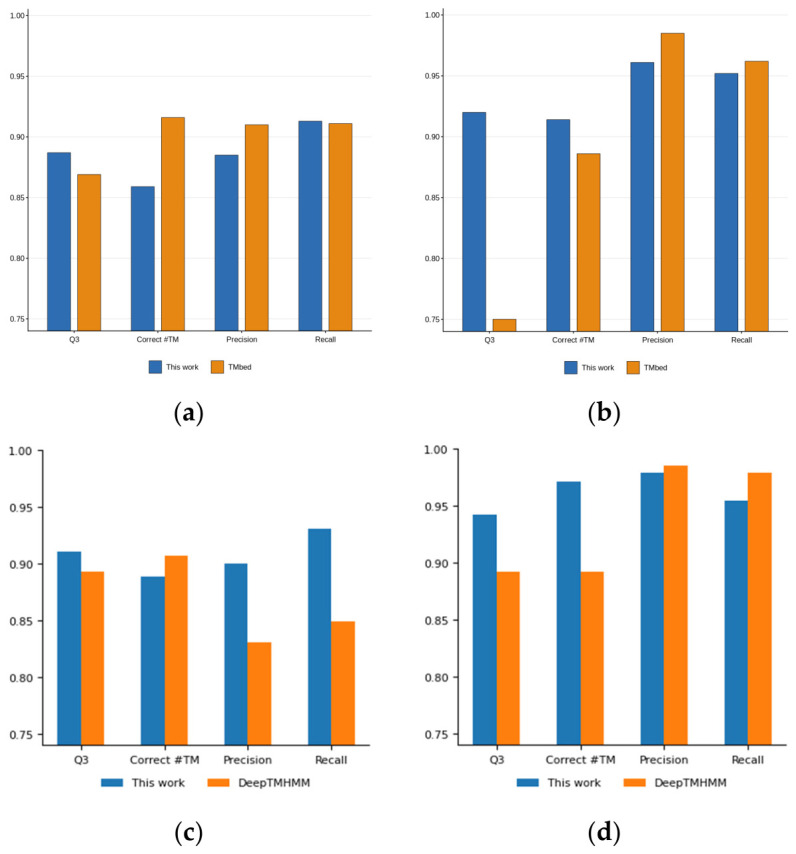
Segment-level comparison of PRED-TMSdeep with TMbed and DeepTMHMM 1.0 on paired common subsets. (**a**,**b**) Comparison with TMbed on 329 α-helical and 48 β-barrel proteins. (**c**,**d**) Comparison with DeepTMHMM 1.0 on 188 α-helical and 48 β-barrel proteins. Metrics are Q3, correct number of transmembrane segments, precision, and recall. Values are shown as fractions, with 1.0 corresponding to 100% performance.

For a direct comparison with existing methods, Figure 5 summarizes the segment-level performance on the common subsets of proteins. For alpha-TMPs, Figure 5a shows that our method performs better in Q3 and slightly better in recall, whereas TMbed performs better in predicting the correct number of transmembrane segments and also shows slightly higher precision. This indicates a trade-off in the alpha-helical subset: PRED-TMSdeep shows stronger residue-level agreement, as reflected by Q3, whereas TMbed more often recovers the exact number of transmembrane segments. For beta-TMPs, Figure 5b shows that our method performs better in Q3 and in predicting the correct number of segments, while TMbed achieves slightly higher precision and recall. The additional DeepTMHMM 1.0 comparison is shown in Figure 5c,d. For alpha-TMPs, Figure 5c shows that PRED-TMSdeep performs better in Q3, precision, and recall, whereas DeepTMHMM 1.0 performs better in predicting the correct number of transmembrane segments. For beta-TMPs, Figure 5d shows that PRED-TMSdeep performs better in Q3 and in predicting the correct number of segments, while DeepTMHMM 1.0 achieves slightly higher precision and recall.

Overall, these results show that the proposed method remains competitive with TMbed and DeepTMHMM 1.0 and preserves its strong segment-level performance, while improving selected metrics depending on the protein class.

### 3.4. Evaluation on Lower-Confidence Transmembrane Segments

We further evaluated the final ensemble model on a lower-confidence test set of transmembrane segments excluded from training because of annotation disagreement between OPM and PDBTM, as summarized in Table 2. These segments were labeled as U and were not used during model development. The ensemble output was obtained by averaging predictions across the five outer-fold models. Precision and recall were computed using the same strict criteria as above, and recall was also evaluated under a relaxed overlap criterion, where any overlap with the reference segment (IoU > 0) was considered correct, to account for annotation uncertainty.

## 4. Discussion

Accurate annotation of membrane and secreted proteins requires resolving several prediction tasks, including transmembrane topology, signal peptide detection, signal peptide type classification, and cleavage-site localization. Existing methods generally address only part of this problem. Topology predictors such as TMbed provide strong membrane-protein annotation at the protein and segment levels but typically treat signal peptides as a single generic category. In contrast, signal peptide predictors such as SignalP distinguish among secretion signal types without reconstructing full transmembrane topology. The results presented here show that PRED-TMSdeep helps bridge this gap by combining both tasks within a single framework and producing more complete whole-protein annotations. The practical gain over previous approaches is therefore not uniform superiority across all metrics, but the ability to provide explicit signal peptide type classification together with competitive transmembrane topology prediction in a single residue-level annotation pipeline.

A central finding of this study is that explicit modeling of signal peptide classes improves signal peptide prediction performance, particularly for the Sec/SPI and Sec/SPII classes. In the comparative analysis, PRED-TMSdeep achieved higher recall for these classes while maintaining low false positive rates, indicating that the gain in sensitivity was not obtained at the expense of reduced specificity. The confidence intervals added in the revised analysis further support the stability of these trends, although modest class-specific differences should be interpreted cautiously when intervals overlap. This supports the view that biologically distinct signal peptide types should be modeled separately rather than merged into a single signal peptide label. Such class-aware modeling appears to help the network capture type-specific sequence characteristics, especially in the N-terminal region, where signal peptides and transmembrane helices may otherwise be easily confused.

The cleavage-site localization results further refine the interpretation of the model’s strengths. PRED-TMSdeep achieved the highest overall top-1 and top-3 cleavage-site accuracy among the compared methods. However, this overall advantage was not uniform across all signal peptide classes. The strongest gains were observed for the predominant Sec/SPI class, with additional favorable point estimates for Sec/SPII, whereas SignalP 6.0 performed better for Tat/SPI cleavage-site localization. For Sec/SPII, PRED-TMSdeep showed favorable point estimates for recall, false positive rate, and cleavage-site localization, although several confidence intervals overlapped and therefore these differences should not be overinterpreted. This pattern is important because it shows that the proposed method improves overall cleavage-site prediction without uniformly dominating at the class level. In practice, the overall improvement remains meaningful because Sec/SPI sequences constitute the largest fraction of the signal peptide data, but the class-specific differences also indicate where further model refinement is needed. This was particularly evident for Tat/SPI, for which we therefore performed an additional class-specific error analysis.

At the transmembrane topology level, PRED-TMSdeep preserved strong performance while expanding the output space to include multiple signal peptide classes. The comparison with TMbed and DeepTMHMM 1.0 showed that topology performance was competitive but metric-dependent, with some metrics favoring PRED-TMSdeep and others favoring the baseline methods. Therefore, we do not interpret the topology results as evidence of uniform superiority over existing topology predictors. Rather, they indicate that the proposed extensions preserve strong topology performance while enabling explicit signal peptide class prediction and cleavage-site localization within the same framework. This is an important result because it indicates that improved signal peptide modeling can be achieved without sacrificing membrane-topology prediction quality. The proposed two-step decoding strategy likely contributes to this balance. By first identifying structured regions under relaxed constraints and then resolving global topology under stricter biological rules, the method can preserve confident local predictions while still enforcing topological consistency at the whole-protein level. In addition, the use of class-specific empirical length constraints in the decoding graph improves biological plausibility and allows the decoder to better reflect the differing sequence characteristics of α-helical segments, β-barrel strands, and distinct signal peptide classes.

Another strength of the present framework is the use of a high-confidence dataset derived from agreement between OPM and PDBTM. Membrane protein annotations are often affected by uncertainty in segment boundaries, and such uncertainty can negatively affect model training and evaluation. By training on regions supported by agreement between two structural annotation resources and reserving lower-confidence segments for separate evaluation, the present study reduces annotation noise during model development while still assessing robustness on more ambiguous cases. This design increases confidence that the observed performance is not simply an artifact of inconsistent training labels.

From a practical perspective, the availability of PRED-TMSdeep as both a web server and a batch command-line tool increases its utility for different use scenarios. The web server facilitates interactive analysis of individual proteins, whereas the command-line implementation supports large-scale annotation and integration into automated computational workflows. In addition, the possibility of incorporating prior topological information in the web server implementation may be useful in expert-guided analyses where partial experimental or biological knowledge is available.

Several limitations should also be acknowledged. First, although PRED-TMSdeep explicitly models three biologically important signal peptide classes, namely Sec/SPI, Sec/SPII, and Tat/SPI, it does not yet cover the full five-class signal peptide scheme supported by SignalP 6.0, which additionally includes Tat/SPII and Sec/SPIII. Thus, while the method improves unified topology and signal peptide annotation for the classes considered here, it does not yet provide a complete solution for the full range of currently recognized signal peptide types.

Second, performance was not uniform across all signal peptide classes, with Tat/SPI cleavage-site localization remaining the clearest class-specific limitation. To investigate this further, we performed an additional Tat/SPI-specific error analysis. The lower Tat/SPI accuracy appears to be driven primarily by the limited number of Tat-related training examples. In the available SignalP training dataset, we counted 398 Tat sequences, whereas our Tat-related set contained only 60 T-labelled sequences. Thus, in each five-fold split, each model was trained on approximately 48 Tat-related examples and evaluated on approximately 12. This limited number likely restricts the ability of the CNN to learn robust Tat-specific cleavage patterns. This interpretation is also consistent with the fact that the same decoding framework performs substantially better for the better-represented Sec/SPI and Sec/SPII classes.

The reduced Tat/SPI cleavage-site accuracy was not mainly explained by systematic confusion with Sec/SPI. Among the 60 T-labelled sequences, 53 were predicted as Tat/SPI and 7 as Sec/SPI. Moreover, class-specific cleavage-site accuracy was calculated only for sequences predicted as the corresponding class; therefore, Tat/SPI sequences predicted as Sec/SPI affected Tat/SPI recall but were not included in the Tat/SPI cleavage-site accuracy calculation. Motif-level analysis further showed that rare or non-canonical cleavage-region motifs were more difficult but did not account for all errors. Among the 60 T-labelled sequences, 38 had AxA-like motifs, 6 had LxG-like motifs, 4 had VxA-like motifs, and 12 had rare or non-canonical motifs. Rare motifs had 5/12 correct predictions within one residue of the annotated cleavage site, whereas AxA-like motifs had 30/38 correct predictions.

The decoder may also contribute indirectly, not because it is different for Tat/SPI, but because it does not explicitly encode motif- or subregion-specific states for any signal peptide class. The Tat/SPI prediction therefore depends on the CNN posterior probabilities together with the allowed signal peptide length range. Since Tat-related examples are scarce, the model may not learn Tat-specific boundary patterns as robustly as for the better-represented signal peptide classes. Future improvements may therefore include increasing the number of Tat/SPI training examples, data augmentation for underrepresented Tat motifs, class- or motif-aware loss weighting, and extending the decoder with Tat-specific subregion or motif-aware states.

Overall, the present results show that joint modeling of transmembrane topology and explicit signal peptide classes is both feasible and beneficial. PRED-TMSdeep improves the completeness of protein sequence annotation by integrating topology prediction, signal peptide classification, and cleavage-site localization within a single biologically constrained framework, while maintaining strong performance in transmembrane protein prediction.

## 5. Conclusions

We developed PRED-TMSdeep, a deep learning framework for the joint prediction of transmembrane topology and signal peptide type in both α-helical and β-barrel proteins. By explicitly distinguishing Sec/SPI, Sec/SPII, and Tat/SPI signal peptides and combining residue-level posterior prediction with a biologically constrained two-step decoding strategy, the method provides more complete whole-protein annotations than approaches that address only topology or only signal peptide classification.

The results show that PRED-TMSdeep preserves strong transmembrane segment and protein-level prediction performance while extending topology prediction to explicit multi-class signal peptide annotation. For signal peptides, PRED-TMSdeep achieved the highest overall top-1 and top-3 cleavage-site localization estimates among the compared methods, mainly reflecting strong performance on the predominant Sec/SPI class and favorable point estimates for Sec/SPII. However, performance was not uniform across all signal peptide classes, and Tat/SPI cleavage-site localization remained weaker than that of SignalP 6.0.

Overall, these findings support the value of unified modeling for membrane and secreted protein annotation, while also indicating that some class-specific cases, particularly Tat/SPI, require further refinement. PRED-TMSdeep therefore offers a practical framework for routine annotation and large-scale analysis, with competitive topology performance and expanded signal peptide classification within a single prediction pipeline. The availability of the method as both a web server and a batch command-line tool further supports its use in proteome-wide studies and reproducible computational workflows.

## Figures and Tables

**Table 1:** Signal peptide prediction performance and cleavage-site localization.

Type	Model	FPR	Recall	Top-k Cleavage Site Accuracy (k = 1)	Top-k Cleavage Site Accuracy (k = 3)
OVERALL (1234 vs. 5059)	PRED-TMSdeep	0.001	0.987	0.892	0.938
	TMbed	0.001	0.988	0.847	0.923
	SignalP 6.0	0.002	0.972	0.862	0.918
Sec/SPI (881)	PRED-TMSdeep	0.006	0.98	0.905	0.95
	SignalP 6.0	0.007	0.931	0.884	0.945
Sec/SPII (293)	PRED-TMSdeep	0.001	0.911	0.97	0.981
	SignalP 6.0	0.005	0.898	0.954	0.958
Tat/SPI (60)	PRED-TMSdeep	0.0	0.883	0.642	0.849
	SignalP 6.0	0.0	0.883	0.849	0.925

Results are reported on the common subset of proteins available for all compared methods. The signal peptide evaluation set contained 1234 SP-positive and 5059 non-SP proteins; class-specific counts are shown in parentheses. Class-specific cleavage-site accuracy was calculated only for true-positive predictions of the corresponding signal peptide class.

**Table 2:** Performance on transmembrane segments excluded from training due to OPM–PDBTM disagreement.

Type	Precision	Recall	Recall (IoU > 0)
alpha-TMPs	0.882	0.852	0.981 (206/210)
beta-TMPs	0.889	0.842	0.947 (18/19)

## Data Availability

The data, source code, pretrained models, and command-line execution instructions presented in this study are openly available in the TMSdeep repository at https://github.com/pbagos/TMSdeep (accessed on 22 June 2026). Publicly available comparative reference data from TMbed were obtained from https://github.com/BernhoferM/TMbed/tree/main/data/cv (accessed on 4 June 2026). Publicly available DeepTMHMM 1.0 cross-validation predictions used for the topology comparison were obtained from https://biolib-public-assets.s3.eu-west-1.amazonaws.com/deeptmhmm/DeepTMHMM.crossval.top (accessed on 4 June 2026). The PRED-TMSdeep software is also available as a web server at https://hannibal.dib.uth.gr/PRED-TMSdeep/ (accessed on 22 June 2026). SignalP 6.0 cross-validation prediction files used for baseline comparisons were obtained from the SignalP authors and are available upon reasonable request and with their permission.

## Supplementary Materials

The following supporting information can be downloaded at: https://www.mdpi.com/article/10.3390/biology15131016/s1, Equation (S1): Definition of the start/end boundary difference criterion used for agreement between OPM and PDBTM annotations; Equation (S2): Definition of the intersection-over-union criterion used for agreement between OPM and PDBTM annotations; Supplementary Text S1: Training and cross-validation; Nested cross-validation procedure; Training details; Requirements and Runtime; Supplementary Table S1: Class-specific signal peptide performance with 95% bootstrap confidence intervals.

## Abbreviations

The following abbreviations are used in this manuscript:

TMP	Transmembrane protein
TM	Transmembrane
SP	Signal peptide
PDB	Protein Data Bank
OPM	Orientation of Proteins in Membranes
PDBTM	Protein Data Bank of Transmembrane Proteins
HMM	Hidden Markov model
CNN	Convolutional neural network
IoU	Intersection over union
SIFTS	Structure Integration with Function, Taxonomy and Sequences
SOV	Segment overlap
Q3	Three-state residue-level accuracy
FPR	False-positive rate
Sec	Secretory pathway
Tat	Twin-arginine translocation
Sec/SPI	Secretory pathway/signal peptidase I
Sec/SPII	Secretory pathway/signal peptidase II
Tat/SPI	Twin-arginine translocation/signal peptidase I
α-helical	alpha-helical
β-barrel	beta-barrel
non-TM	Non-transmembrane
