# Supplementary material for: PRED-TMSdeep: Prediction of Transmembrane Topology and Signal Peptides Using Deep Learning
Source: Biology (Basel). 2026 Jun 26;15(13):1016. doi: 10.3390/biology15131016 (PMC13359764; doi:10.3390/biology15131016)
Supplement: Supplementary file 1 [file biology-15-01016-s001.zip › biology-4319744-supplementary.pdf]

Supplementary Material

PRED-TMSdeep: Prediction of transmembrane topology and signal peptides using deep learning

Grigorios A. Moschos, Konstantinos D. Tsirigos, Ioannis A. Tamposis, Pantelis G. Bagos

Contents

Data collection and preprocessing 2

Training and cross-validation 2

Statistical analysis 3

Requirements and Runtime 3

References 4

Data collection and preprocessing

Equations S1, S2

TMbed [1] introduced these criteria for evaluation; here we use Equations (S1)–(S2) to define high-confidence TM segments

(1) **Boundary agreement**: the start and end positions of the corresponding segments differed by no more than five residues:

$$max(|S_{OPM} - S_{PDBTMM}|, |E_{OPM} - E_{PDBTMM}|) \leq 5 \tag{S1}$$

where S_{OPM} , S_{PDBTMM} denote the start positions, and E_{OPM} , E_{PDBTMM} the end positions of the corresponding TM segments from OPM [2] and PDBTM [3], respectively.

(2) The **Intersection-over-Union (IoU)** between the two segment annotations must be at least 0.5

$$\frac{max(0, min(E_{OPM}, E_{PDBTMM}) - max(S_{OPM}, S_{PDBTMM}) + 1)}{max(E_{OPM}, E_{PDBTMM}) - min(S_{OPM}, S_{PDBTMM}) + 1} \geq 0.5 \tag{S2}$$

Training and cross-validation

Text S1

We adopted the nested cross-validation protocol used in TMbed to ensure robust model evaluation and hyperparameter tuning.

Dataset stratification and fold assignment

To ensure balanced representation across protein types and signal peptide classes, the dataset was stratified into ten distinct groups based on two factors:
(i) Protein class/type: α-helical TMPs (further divided into single-pass and multi-pass), β-barrel TMPs, and non-transmembrane proteins.
(ii) Signal peptide presence: with or without SP, plus separate groups for LIPO and TAT.
This stratification produced ten groups in total, which were then randomly and evenly distributed across five folds for cross-validation.

Nested cross-validation procedure

We followed TMbed's nested cross-validation protocol. In each outer split, one-fold was held out as an **independent test set** that was never used during model development. The remaining four folds were further split 3–1 into training and validation sets for hyperparameter selection. The number of training epochs was determined by monitoring validation loss, after which the model was retrained on the combined training and validation data for the selected epoch count and evaluated on the independent test fold. This procedure was applied to both the primary joint model and the β-barrel refinement model. Importantly, the same outer test-fold assignment was used for both models in each split, yielding paired models per fold (primary joint model; β-barrel refinement model trained only on the β-barrel subset of that fold). Final performance metrics were computed by aggregating predictions across all five independent test folds, without reporting per-fold statistics.

Training details

Primary joint model. One model was trained per outer fold (five total). We used AdamW [4] (β_1 = 0.9, β_2 = 0.999) with decoupled weight decay of 0.1, batch size 16, and a learning rate starting at 0.01 with an exponential decay applied once per epoch (decay rate 0.8, staircase schedule). Each outer-fold model was trained for 11, 12, 11, 12, and 13 epochs for folds 1–5, respectively.

β-barrel refinement model. This network used the same optimizer settings (AdamW; β_1 = 0.9, β_2 = 0.999; weight decay 0.1) and the same staircase learning rate scheme but with a milder decay (starting LR 0.01; decay rate 0.97 per epoch). Training was full batch (batch size equal to the number of training sequences in the fold). The CNN capacity was reduced to 32 channels per layer (64 in TMbed and in our primary model). We also applied an additional input-dropout of 0.5 to the transformer embeddings. Five refinement models were trained (one per outer fold), each using only the β-barrel sequences from that fold's train/validation split (no α-helical or non-TM proteins); each model trained for 83 epochs and was evaluated on the β-barrel subset of the held-out outer test fold.

Statistical analysis

Supplementary Table S1. Class-specific signal peptide performance with 95% bootstrap confidence intervals.

Class	Method	n	Recall (95% CI)	FPR (95% CI)	Top-1 CS accuracy (95% CI)	Top-3 CS accuracy (95% CI)
Sec/SPI	PRED-TMSdeep	881	0.980 [0.970, 0.989]	0.006 [0.004, 0.009]	0.905 [0.885, 0.924]	0.950 [0.935, 0.964]
Sec/SPI	SignalP 6.0	881	0.931 [0.913, 0.947]	0.007 [0.005, 0.010]	0.884 [0.862, 0.905]	0.945 [0.930, 0.960]
Sec/SPII	PRED-TMSdeep	293	0.911 [0.877, 0.942]	0.001 [0.0005, 0.0023]	0.970 [0.949, 0.989]	0.981 [0.964, 0.996]
Sec/SPII	SignalP 6.0	293	0.898 [0.862, 0.931]	0.005 [0.004, 0.007]	0.954 [0.926, 0.977]	0.958 [0.933, 0.980]
Tat/SPI	PRED-TMSdeep	60	0.883 [0.793, 0.957]	0.000 [0.000, 0.000]	0.642 [0.508, 0.774]	0.849 [0.745, 0.940]
Tat/SPI	SignalP 6.0	60	0.883 [0.797, 0.960]	0.000 [0.000, 0.001]	0.849 [0.745, 0.938]	0.925 [0.846, 0.983]
Total SP	PRED-TMSdeep	1234	0.987 [0.981, 0.993]	0.001 [0.0004, 0.0025]	0.892 [0.874, 0.909]	0.938 [0.924, 0.952]
Total SP	TMbed	1234	0.988 [0.978, 0.996]	0.001 [0.0004, 0.0020]	0.847 [0.825, 0.875]	0.923 [0.900, 0.945]
Total SP	SignalP 6.0	1234	0.972 [0.963, 0.981]	0.002 [0.001, 0.004]	0.862 [0.843, 0.881]	0.918 [0.902, 0.934]

Note. CS, cleavage site; FPR, false positive rate. For consistency with the PRED-TMSdeep label space, SignalP 6.0 Tat/SPI and Tat/SPII predictions were merged into the Tat/SPI-related class. Class-specific cleavage-site accuracy was calculated only for true-positive predictions of the corresponding class. TMbed is reported only for Total SP because it predicts a generic signal peptide class rather than separate Sec/SPI, Sec/SPII, and Tat/SPI classes.

Requirements and Runtime

PRED-TMSdeep has computational requirements comparable to TMbed because both methods rely on the same protein language model embedding framework, which represents the main computational cost in terms of runtime and memory usage. The additional steps introduced in PRED-TMSdeep, namely the two-step Viterbi decoding procedure instead of a single decoding pass and the beta-barrel refinement stage, make the pipeline slightly slower than TMbed. However, these steps operate after the embedding and CNN prediction stages and do not substantially affect the overall computational feasibility of the method.

The beta-barrel refinement model is applied only to proteins classified as beta-barrel candidates, so it does not add overhead for most input sequences. In practical testing, PRED-TMSdeep processed approximately 6,000 protein sequences in about 15 minutes on a Google Colab A100 GPU. Therefore, PRED-TMSdeep can be used for proteome-scale prediction in a manner comparable to TMbed, with the main resource requirement remaining the generation and processing of protein language model embeddings.

References

1. Bernhofer M, Rost B. TMbed: transmembrane proteins predicted through language model embeddings. BMC Bioinformatics [Internet]. 2022 [cited 2025 July 14];23(1). https://doi.org/10.1186/s12859-022-04873-x

2. Lomize MA, Pogozheva ID, Joo H, Mosberg HI, Lomize AL. OPM database and PPM web server: resources for positioning of proteins in membranes. Nucleic Acids Research. 2012;40(D1):D370–6. ψ

3. Kozma D, Simon I, Tusnády GE. PDBTM: Protein Data Bank of transmembrane proteins after 8 years. Nucleic Acids Research. 2012;41(D1):D524–9. https://doi.org/10.1093/nar/gks1169

4. Loshchilov I, Hutter F. Decoupled Weight Decay Regularization [Internet]. arXiv; 2017 [cited 2025 Sept 25]. https://doi.org/10.48550/ARXIV.1711.05101
